# cMR in acute myocardial infarction: correlation between myocardial scar and echocardiographic strain

**DOI:** 10.1186/1532-429X-14-S1-P25

**Published:** 2012-02-01

**Authors:** Gaby Weissman, Michael Kern, Ana Barac, Manuel A Gonzalez, Rebecca Torguson, Ron Waksman, Anthon Fuisz

**Affiliations:** 1 Washington Hospital Center, Washington, DC, USA; 2 Case Western Reserve University, Cleveland, OH, USA

## Summary

Global longitudinal and circumferential strain was assessed by echo in 15 patients after acute MI. MRI parameters including ventricular volumes, scar, and area at risk were assessed 3+/-2 days post STEMI and at 30 day follow up. Echo strain measures correlated most strongly with LVEF (r=0.81 and 0.88 longitudinal ad circumferential strain, p<0.0001) and infarct size (r=-0.68 and r=-0.69, p<0.001). There were moderate correlations between longitudinal strain and change in LVEDV (r=-0.61, p<0.05).

## Background

Cardiac MR (CMR) is a powerful tool in the evaluation myocardial scar and volumes. Speckle tracking by echo is used to evaluate myocardial strain. The correlation between strain measures and MR measures of scar is not well defined.

### Hypothesis

CMR parameters, area at risk (AAR), infarct size (IS), and salvage area (SA), correlate with global echo strain in patients with a ST elevation MI (STEMI).

## Methods

Contrast enhanced(CE) CMR and 2-D echocardiography imaging were performed in patients within 3+/-2 days post STEMI. CMR-derived IS was measured from the late CE images and AAR was measured from T2 black blood images. SA was calculated as AAR/IS. Global longitudinal (LS) and circumferential (CS) strain were assessed from standard apical (16 segment model for LS) or midventricular short axis views using 2-D speckle-tracking software (2D CPA, TomTec, Germany).

## Results

15 patients with STEMI completed the protocol. Age was 50.8+/-12.6 years (86.7% male). Baseline LVEDV was 203.4 +/- 51.7ml and EF was 51.1% +/- 14.7% with follow up LVEDV 219.9 +/- 72.2ml (p=0.15) and EF 50.8% +/- 13.8% (p=0.8). Average IS was 16.4+/-10.4%, AAR was 23.4+/-12.2%, and SA was 7.0+/-5.7% at baseline. Baseline LS was -13.5 +/- 4.1 and CS was -19.4 +/- 6.3. Global longitudinal strain was associated with peak troponin I levels (r=-0.74, P<0.001). Correlating LS and CS to baseline MRI parameters showed a strong correlation to LVEF (r=0.81 and 0.88 respectively, p<0.0001) and IS (r=-0.68 and r=-0.69, p<0.001). There was moderate correlation of LS and CS to LVEDV (r=-0.55 and -0.57), p<0.05) and AAR (r=-0.61 and r=-0.57, p<0.05). There was no correlation between strain and SA. There were modest correlations between initial LS and change in LVEDV but not with CS and change in LVEDV (r=-0.61, p<0.05 and r=-0.48, p=0.07).

## Conclusions

Echo strain measurements correlated most strongly with LVEF and IS. There were modest associations between strain at baseline and early LV remodeling.

## Funding

None.

**Figure 1:**
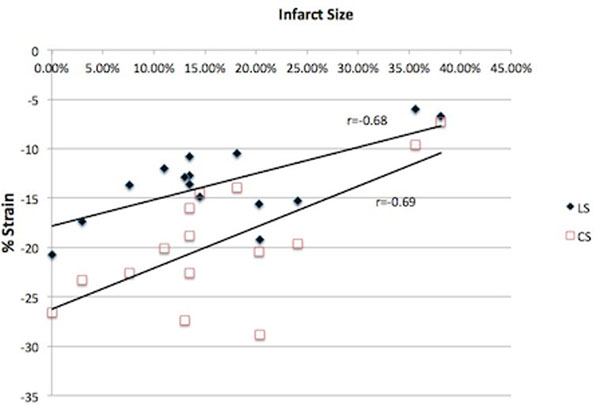
Correlation between infarct size and longitudinal (LS) and circumferential (CS) strain.

